# Exploring the Role of Circular RNA in Bone Biology: A Comprehensive Review

**DOI:** 10.3390/cells13120999

**Published:** 2024-06-07

**Authors:** Maria Teresa Valenti, Roberta Zerlotin, Mattia Cominacini, Silvia Bolognin, Maria Grano, Luca Dalle Carbonare

**Affiliations:** 1 Department of Neurosciences, Biomedicine and Movement Sciences, University of Verona, 37100 Verona, Italy; 2 Department of Precision and Regenerative Medicine and Ionian Area, University of Bari, 70124 Bari, Italy; roberta.zerlotin@uniba.it (R.Z.); maria.grano@uniba.it (M.G.); 3 Department of Engineering for the Innovation Medicine, University of Verona, 37100 Verona, Italy; mattia.cominacini@univr.it (M.C.); luca.dallecarbonare@univr.it (L.D.C.); 4 MERLN Institute, Maastricht University, Universiteitssingel 40, 6229 ET Maastricht, The Netherlands; silvia.bolognin@maastrichtuniversity.nl

**Keywords:** bone, circular RNAs, osteogenesis, differentiation

## Abstract

Circular RNAs (circRNAs) have emerged as pivotal regulators of gene expression with diverse roles in various biological processes. In recent years, research into circRNAs’ involvement in bone biology has gained significant attention, unveiling their potential as novel regulators and biomarkers in bone-related disorders and diseases. CircRNAs, characterized by their closed-loop structure, exhibit stability and resistance to degradation, underscoring their functional significance. In bone tissue, circRNAs are involved in critical processes such as osteogenic differentiation, osteoclastogenesis, and bone remodeling through intricate molecular mechanisms including microRNA regulation. Dysregulated circRNAs are associated with various bone disorders, suggesting their potential as diagnostic and prognostic biomarkers. The therapeutic targeting of these circRNAs holds promise for addressing bone-related conditions, offering new perspectives for precision medicine. Thus, circRNAs constitute integral components of bone regulatory networks, impacting both physiological bone homeostasis and pathological conditions. This review provides a comprehensive overview of circRNAs in bone biology, emphasizing their regulatory mechanisms, functional implications, and therapeutic potential.

## 1. Introduction

Bone homeostasis, the balance between bone formation and resorption, is mainly regulated by osteoblasts and osteoclasts [1]. Osteoblasts originate from bone marrow mesenchymal stem cells (BMSCs) and are bone-forming cells while osteoclasts derive from monocyte/macrophage lineage and are bone-resorption cells [2]. Circular RNAs (circRNAs) have emerged as significant players in the regulation of gene expression, even if their involvement in various biological processes continues to be unraveled. In recent years, research into the role of circRNAs in bone biology has garnered significant attention, shedding light on their potential as novel regulators and biomarkers in bone-related disorders [3].

CircRNAs are a class of non-coding RNAs characterized by a covalently closed loop structure, making them stable and resistant to exonucleases [4]. This stability is attributed to the absence of free ends that are susceptible to degradation, making circRNAs resilient to the enzymatic degradation processes that target linear RNAs [4]. Consequently, circRNAs can function as robust regulators of gene expression, exerting their effects over extended periods without being rapidly degraded [4]. Their abundance and conservation across species underscore their functional significance [5]. In particular, the functional roles of circRNAs have been reported in cattle, goats, and sheep, with a specific focus on their influence on milk yield, meat quality, and muscle growth, as well on wool production [5]

In bone tissue, circRNAs have been implicated in diverse cellular processes, including osteogenic differentiation, osteoclastogenesis, and bone remodeling [6]. Through various molecular mechanisms, such as microRNA sponging, interaction with RNA-binding proteins, and the modulation of gene transcription, circRNAs exert intricate regulatory control over key signaling pathways involved in bone homeostasis [6].

Thus, circRNAs play pivotal roles in promoting bone development and maintenance [6,7,8]. Conversely, certain circRNAs have been associated with osteoclastogenesis and bone resorption, emphasizing their dual roles in bone metabolism [6,9,10]. Furthermore, the dysregulation of circRNAs has been associated with various bone disorders, including osteoporosis, osteoarthritis, osteosarcoma, and bone metastasis [9,11,12,13,14,15,16,17,18,19,20,21]. Importantly, profiling studies have identified distinct circRNA expression patterns in diseased bone tissues compared to healthy controls, suggesting their potential utility as diagnostic and prognostic biomarkers [22,23,24]. Moreover, the therapeutic targeting of dysregulated circRNAs holds promise for the development of novel interventions for bone-related disorders, offering innovative approaches to precision medicine.

In conclusion, circRNAs represent an integral component of the regulatory network governing bone biology, with implications for both physiological bone homeostasis and pathological conditions. Despite significant progress, several challenges remain, including elucidating the precise mechanisms of circRNA activity and the tissue-specific functions, as well the development of efficient therapeutic strategies. Nevertheless, new research aiming to explore the role of circRNAs in bone biology holds immense potential for advancing the understanding of skeletal health and disease, paving the way for innovative diagnostic and therapeutic approaches in the field of bone disorders. This review aims to provide a comprehensive overview of the current understanding of circRNAs in bone biology, highlighting their regulatory mechanisms, functional implications, and therapeutic potentials.

## 2. Circular RNAs (circRNAs)

Circular RNAs (circRNAs) are non-coding RNAs characterized by a circular structure. Unlike linear RNAs, circRNAs form covalently closed loops, devoid of 5′ caps and 3′ polyadenylated tails. This circularization is generally generated by back-splicing, the process by which a downstream splice donor site joins with an upstream splice acceptor site, resulting in a circular RNA molecule. In particular, during the process of back-splicing, the 3′ end of an exon is linked to the 5′ end of the same exon or an upstream one, producing a 3′,5′-phosphodiester bond. This 3′,5′-phosphodiester bond forms a closed-loop structure characterized by a back-splicing junction site, which acts as a hallmark of circRNAs [25,26]. Furthermore, circular RNA can also indirectly arise from small nuclear RNAs (snRNAs), mitochondrial RNAs, ribosomal RNAs (rRNAs), and transfer RNAs (tRNAs) through intron self-splicing processes [27]. Thus, by using RNA sequencing (RNA-seq) technologies and computational methodologies for circular RNA annotation, it has been reported that circRNAs can originate from exons, introns, 5′ untranslated regions (UTRs), 3′ UTRs, or antisense sequences, and can be categorized into exon-derived circRNA (ecRNA), intron-derived circRNA (ciRNA), exon-intron circRNA (EIcircRNA), and others; these have been identified across a spectrum of organisms, including viruses, archaea, plants, parasites, and mammals [27,28]. ecRNAs are the most abundant form of circRNAs and originate exclusively from exons. They can include one or more exons from the original gene. The ecRNAs mainly function as molecular sponges for microRNAs (miRNAs). By binding to miRNAs, ecRNAs can prevent miRNAs from interacting with their target mRNAs, thereby modulating gene expression post-transcriptionally [29,30].

The ciRNAs come from introns, the non-coding sequences within pre-mRNA. ciRNAs generally reside in the nucleus, acting as transcriptional regulators. In fact, ciRNAs can increase the transcription of their parent genes by interacting with the RNA polymerase II complex or other molecules involved in the transcription process, thereby promoting efficient gene expression [31,32].

EIciRNAs contain both exons and introns. Similar to ciRNAs, EIciRNAs are involved in the transcription process. Within the nucleus, EIciRNAs can perform regulatory functions, such as interacting with U1 snRNA through specific RNA–RNA interactions. This interaction forms a complex that may be further associated with the Pol II transcription complex at the promoters of parental genes to enhance gene expression [33].

Thanks to this circular structure, circRNAs have a remarkable stability and resistance to exonucleases, enhancing their longevity within the cell compared to their linear counterparts. Indeed, the stability conferred by their circular structure enables circRNAs to persist in the cell for extended periods, allowing for prolonged regulatory activity. Since circRNAs lack free 5′ or 3′ ends, endoribonucleolytic cleavage appears to be the primary mechanism for circRNA degradation. Notably, Liu et al. [34] recently reported the discovery of a circRNA endonuclease, RNase L, which is broadly able to degrade circRNAs. Furthermore, it has been demonstrated that m6A can facilitate the degradation of both mRNAs and circRNAs [35]. Moreover, circRNAs exhibit remarkable abundance and conservation across species, further underscoring their functional significance. High-throughput sequencing studies have revealed a plethora of circRNAs expressed in various cell types and tissues, indicating the widespread transcription of these molecules throughout the genome [36,37,38]. Additionally, circRNAs have been found to be evolutionarily conserved across different species, from plants to animals, suggesting their importance in fundamental biological processes. Interestingly, it has been demonstrated that the total number of circRNAs significantly increases from worms and fruit flies to mice and humans, suggesting an increase in circRNA expression during evolution [39].

The abundance and conservation of circRNAs suggest their potential as crucial regulators of gene expression and cellular processes. Indeed, recent studies have revealed the multifaceted roles of circRNAs in various biological contexts, such as cell proliferation, differentiation, and development. These regulatory functions are mediated through diverse mechanisms, including serving as microRNA sponges, interacting with RNA-binding proteins, modulating alternative splicing, and regulating transcription and translation [40,41,42].

### 2.1. circRNAs in Bone Development and Maintenance

It has been reported that specific circRNAs exert pivotal regulatory functions in various aspects of bone development and maintenance. Notably, the depletion or dysregulation of certain circRNAs has been associated with aberrant bone phenotypes, highlighting their significance in orchestrating key signaling pathways during the osteogenic process. In particular, circRNAs have been found to regulate the osteogenic differentiation of mesenchymal stem cells (MSCs), which are multipotent cells capable of differentiating into various cell types, including osteoblasts (bone-forming cells) [8,43,44,45,46]. Indeed, circRNAs regulate the expression of target genes involved in osteogenic differentiation via sponging microRNAs (miRNAs). CircFOX1 plays a significant role in maintaining the stemness of mesenchymal cells and may be involved in the osteogenic differentiation process [47,48]. It has been demonstrated that circFOX1 increases during the period of osteogenesis from 3 to 14 days [48]. This increase could indicate a significant role of circFOX1 in regulating the osteogenic differentiation of mesenchymal cells. The maintenance of its expression may be related to the need to keep the cells in a progenitor state during the period of differentiation. In addition, CircFOXP1 promotes the osteogenic capabilities of human adipose stem cells (hASCs) by sequestering miR-33a-5p. Conversely, miR-33a-5p reduces osteogenesis by specifically targeting the 3′-UTR region of the *FOXP1* gene, thereby reducing *FOXP1* expression. These results suggest that circFOXP1/ miR-33a-5p axis plays a central role in regulating the differentiation of hASCs into osteogenic lineage cells through the modulation of *FOXP1* expression [43]. Moreover, the hsa_circ_0006766 molecule appears to play a significant regulatory role in orchestrating the osteogenic differentiation process of human bone-marrow-derived mesenchymal stem cells (hBM-MSCs) through a regulatory axis involving hsa_circ_0006766, miR-4739, and Notch2 [44]. In particular, during the 7-day induction of osteogenic differentiation, hsa_circ_0006766 expression in hBM-MSCs significantly increased compared to day 0 [44]. In addition, Circ_0067680 expression increases after 7 and 14 days of osteogenic differentiation, enhancing the osteogenic differentiation of hBMSCs via the miR-4429/CTNNB1/Wnt/β-catenin signaling pathway [45], and circRNA422 promotes the proliferation and osteogenic differentiation of bone marrow mesenchymal stem cells (BMSCs) by modulating the expression of *SP7* and *LRP5* [49]. Conversely, circHGF could be regarded as a negative regulator of osteogenic differentiation. Indeed, it has been demonstrated that circHGF inhibits the proliferation and osteogenic differentiation of BMSCs in osteonecrosis of the femoral head (ONFH) by targeting the miR-25-3p/SMAD7 axis [50], and CircPOMT1 and circMCM3AP impair the osteogenic differentiation process of human adipose-derived stem cells by specifically targeting the miR-6881-3p/BMPs signaling pathway [51]. As SMAD7 and BMPs are involved in the early phases of osteogenesis, it is evident that circHGF, CircPOMT1, and circMCM3AP mainly hinder the early stages of osteogenesis. However, in recent years, numerous other circular RNAs were discovered to contribute to the process of osteogenic differentiation (Table 1).

CircRNAs also play roles in maintaining bone homeostasis by regulating bone metabolism and the remodeling processes. For example, circRNA_ circ_0006859 was found to inhibit osteogenesis and promote adipogenesis in human in human bone marrow mesenchymal stem cells by targeting the signaling miR-431-5p/Rho-associated protein kinase 1 (ROCK1) [67] and circRNA-CDR1 promotes adipogenesis and inhibits osteogenic differentiation in osteonecrosis of the femoral head [68].

Additionally, several circRNAs have been shown to be involved in promoting osteoclastogenesis and bone resorption, highlighting their roles in bone metabolism. Osteoclastogenesis is the process by which osteoclast precursor cells differentiate into mature osteoclasts, which are responsible for bone resorption, and it is well known that the osteoclasts’ differentiation from precursor cells is tightly regulated [69]. Thus, by regulating key signaling pathways involved in this process, circRNAs have been implicated in modulating osteoclastogenesis. For instance, Deng et al. reported that CircZNF367 promotes osteoclasogenesis and can contribute to osteoporosis through the interaction with FUS, maintaining the stability of *CRY2* mRNA and thus enhancing proliferation and TRAP, NFATc1, and c-FOS expression in osteoclast [70]. Tang et al. have demonstrated that circ_0029463 drives osteoclast differentiation through the modulation of the miR-134-5p/Rab27a axis and promoting RANKL-induced differentiation [10]. In addition, circRNA_009934, whose expression increases in mature osteoclasts, triggers osteoclast-mediated bone resorption by suppressing miR-5107; circFam190a, increasing from the early to middle to late stages of osteoclastogenesis, emerges as a pivotal inducer of osteoclast differentiation by amplifying the AKT1/HSP90β complex; and, recently, exosomal circ_0000722, originating from periodontal ligament stem cells undergoing osteogenic differentiation, has been shown to stimulate osteoclastogenesis in RAW264.7 cells [71,72,73].

These findings collectively highlight the intricate regulatory roles of circRNAs in bone metabolism, wherein specific circRNAs modulate key signaling pathways in cellular processes, such as osteoblast differentiation, bone formation, and osteoclastogenesis. Consequently, the dysregulation of bone-related circRNAs can impair the balance between bone formation and resorption, leading to skeletal abnormalities and bone-related disorders.

### 2.2. circRNAs in Bone Disorders

Understanding the regulatory mechanisms exerted by circRNAs in bone biology not only enhances the knowledge of skeletal physiology but also could suggest potential targets and therapeutic implications for the treatment of bone disorders and diseases. Profiling studies have identified distinct circRNA expression patterns in pathological bone tissues compared to healthy controls, offering insights into disease mechanisms and potential therapeutic targets [22]. Indeed, dysregulation of most of the aforementioned circRNAs associated with osteogenesis or osteoclastogenesis is involved in the pathogenesis of skeletal diseases [9,55,56,67,68,70]. However, research in recent years has significantly increased the number of identified circRNAs associated with skeletal pathologies and many other circRNA molecules are continually being discovered. Below, we report a few of them.

Osteoporosis: Osteoporosis, a common skeletal disorder characterized by reduced bone density and increased susceptibility to fractures, represents a significant public health concern, particularly among aging populations. In recent years, circRNAs have been reported to be involved in the pathogenesis of osteoporosis.

Among the circRNA expression patterns, circ-VANGL1 has been found to be upregulated in osteoporotic patients and it has been demonstrated that this circRNA contributes to the progression of osteoporosis by acting as a sponge for miRNA-217, thereby modulating the expression of RUNX2 [74]. Conversely, circ_0027885 has the potential to sequester miR-203-3p, thus controlling the expression of RUNX2 and reducing the progression of osteoporosis [75]. It has been reported that circ_0006873 and circ_0002060 expression levels are associated with low bone mineral density (BMD) in patients with osteoporosis and that circ_0002060 shows promising diagnostic utility for osteoporosis [76]. In addition, circRNA-0076906 and circRNA-0134944 dysregulation is associated with the onset of osteoporosis, as well as an increase in osteoporotic fractures among postmenopausal females [77].

Additionally, circulating circRNAs present in bodily fluids such as blood or urine could serve as non-invasive biomarkers for monitoring disease progression or response to treatment [78,79]. Zhao et al., by performing circRNA microarray profiling and validating their findings through qRT-PCR, identified five upregulated circRNAs (hsa_circ_0028882, hsa_circ_0001275, hsa_circ_0006766, hsa_circ_0007788, and hsa_circ_0003391), and one downregulated circRNA (hsa_circ_0006801), in peripheral blood mononuclear cells (PBMCs) of postmenopausal osteoporosis (PMOP) patients and, among these circRNAs, only hsa_circ_0001275 exhibited significant differential expression compared to controls [80]. The expression of circ_HECW2 in serum samples of osteoporosis (OP) patients showed heightened levels, indicating diagnostic significance in osteoporosis cases. In fact, it has been demonstrated that circ_HECW2 acts by reducing the levels of mature miR-1224-5p through binding to pre-miR-1224, consequently upregulating PDK2 and promoting osteoblast apoptosis [81]. Additionally, a negative correlation was observed between circ_HECW2 expression and lumbar T-score and circ_HECW2 levels correlate with the severity of the disease [81]. Further research elucidating the specific functions and regulatory networks of circRNAs in bone metabolism can suggest novel therapeutic targets and strategies for managing bone-related conditions.

Osteosarcoma: Osteosarcoma arises from mesenchymal stem cells and is considered the most common primary malignant bone tumor, particularly in adolescent subjects [82]. Typically osteosarcoma is localized in the femur and tibia and shows a marked aggressiveness with a high tendency to generate lung metastasis [83]. Despite advancements in prognosis through chemotherapy or chirurgic approaches, the survival possibility for metastatic patients is poor [84]. Thus, the identification of novel biomarkers and in-depth understanding of the molecular mechanisms to pinpoint potential therapeutic targets for osteosarcoma is an important challenge. Extensive research has been conducted on genetic biomarkers in osteosarcoma, with circular RNAs (circRNAs) emerging as significant players in its development [85,86]. Previous studies have reported that circXPO1, upregulating the expression of XPO1 by sponging multiple miRNAs, and circKCNH1, modulating the miR-1225-3p/KCNH1 axis, can promote the proliferation of osteosarcoma by regulating their related genes, suggesting a positive correlation between the expression of circRNAs and that of their associated genes [87,88]. Circ_0001821 promotes osteosarcoma invasion by suppressing miR-205-5p, miR-526b, and miR-26b-5p, thus leading to the upregulation of *c-FLIP*, *FOXC2*, and *CCNB1*, respectively [89,90,91]. Additionally, circ_0005721, which acts by upregulating *TEP1* via miR-16-5p, can be detected in the peripheral blood and shows a close association with osteosarcoma [92,93]. Importantly, it has been suggested that this circRNA could potentially be considered a minimally invasive biomarker to distinguish osteosarcoma from healthy individuals and from patients with benign bone pathologies [86,92]. It has also been reported that circ_0001721 can modulate the expression of *GAB1*, *MAPK7*, and *E2F2* by acting as a sponge for miR-520a-3p, miR-372-3p, and miR-198, thus promoting osteosarcoma progression [86,94]. It is important to know that a particular miRNA can be targeted by multiple circRNAs, thus influencing target RNAs expression [86]. Circ_0017311, hsa_circ_0000006, and hsa_circ_0000253 can regulate LDHA, VEGF, and TGFβ2 by sponging miR-578, thus increasing osteosarcoma progression [95,96]. In addition to the role of miRNA sponging, circRNAs can act by interacting with proteins. For instance, circECE1 is able to regulate the energetic metabolism of osteosarcoma by interacting with the c-Myc protein [97].

Hereditary bone diseases: Different groups of genetic disorders affect skeletal development, structure, and function [98,99]. These hereditary conditions often arise from mutations in genes encoding proteins crucial for bone formation, mineralization, or remodeling [100,101]. While research into the role of circRNAs in various biological processes is still ongoing, emerging evidence suggests their involvement in the pathogenesis of hereditary bone diseases [102]. Studies investigating the molecular mechanisms underlying hereditary bone diseases have identified dysregulated gene expression and signaling pathways as key contributors to skeletal abnormalities. However, at present, no study has focused on understanding the involvement of circRNAs in hereditary bone pathologies such as osteogenesis imperfecta, osteopetrosis, and skeletal dysplasias. Thus, given their regulatory roles in gene expression, circRNAs could be considered a promising target when studying these pathologies. Nonetheless, the emerging field of circRNA biology holds significant potential for advancing our understanding of hereditary bone diseases and may pave the way for novel diagnostic and therapeutic strategies in the future.

## 3. Animal Models

Animal models play a crucial role in advancing our understanding of bone biology and the pathophysiology of bone-related diseases. These models, ranging from zebrafish to larger mammals, allow researchers to investigate the complex molecular mechanisms underlying bone development, remodeling, and disease progression in a controlled environment [103,104,105,106]. Thus, animal models, particularly murine models, have been used to investigate the expression patterns, regulatory functions, and therapeutic potentials of circRNAs in bone tissue, shedding light on their roles in bone physiology and pathology [7,71,107,108,109,110,111,112,113,114,115].

By using ovariectomy (OVX) in mice, it was demonstrated that the knocking down of circRNA_28313 led to a reduction in bone resorption [108]. Furthermore, an increased expression of circ-SLC8A1 has been observed in OVX mice. Moreover, administering circHmbox1-CH to OVX mice significantly improved trabecular bone structures, suggesting a promising therapeutic approach involving increasing circHmbox1 levels for osteoporosis treatment [112,114]. By using a rat model, it was demonstrated that the circRNA_016717/miR-501-5p/Sfrp1 axis plays an important role in the protective activity of diosgenin against alveolar bone loss [109]. Furthermore, a glucocorticoid-induced osteoporosis (GIOP) rat model was utilized to demonstrate the association of circARSB, circAKT3, circPTEN, and circTRPM7 with osteogenic differentiation through a circRNA-targeted miRNA–mRNA axis [116]. This insight could shed light on the pathophysiological mechanism of GIOP.

Through the generation of a rat model for bone cancer pain, it has been shown that circStrn3 plays a role in regulating pain associated with bone cancer [117]. Specifically, the rat model was established through the inoculation of rats with Walker 256 cells, leading to noticeable ascites symptoms within 9–12 days post-inoculation. To assess sensitivity to stimuli, the von Frey hair test, involving the application of calibrated nylon filaments of different forces to the skin, was employed [117]. Furthermore, enhanced healing in rats with induced calvarial defects was obtained through the implantation of cell-overexpressing circ-CTTN hydrogels [118].

Thus, by comparing the circRNA expression profiles of healthy and pathological animal models, researchers can identify candidate circRNAs involved in the pathogenesis and progression of diseases. This approach not only enhances our understanding of the molecular mechanisms underlying bone disease but also offers potential targets for therapeutic intervention.

Furthermore, animal models provide a platform for assaying the diagnostic and prognostic utility of circRNAs in bone disorders. By correlating circRNA expression levels with disease severity, bone mineral density, and fracture risk in animal models, it is possible to assess the clinical relevance of circRNAs as biomarkers for bone health and disease progression. This translational approach facilitates the validation of circRNA-based diagnostic assays and prognostic indicators in preclinical settings before clinical application in human patients.

In summary, animal models serve as useful tools for investigating the role of circRNAs in bone biology and pathology. By leveraging these models, researchers can elucidate the functional significance of circRNAs in the bone metabolism, disease pathogenesis, and therapeutic responses, ultimately paving the way for the development of circRNA-based diagnostic and therapeutic strategies for bone disorders.

## 4. Therapeutic Strategies Targeting circRNAs in Bone Disorders

Several therapeutic approaches have been explored to modulate circRNA expression levels or activity for the treatment of different pathologies. These approaches include circRNA overexpression using viral vectors (Lentiviral and adenoviral vectors) or synthetic delivery systems (by direct synthesis and purification of circRNAs), the knockdown of specific circRNAs using antisense oligonucleotides or RNA interference, and the manipulation of circRNA-associated signaling pathways using small molecule inhibitors or gene editing technologies (using the CRISPR/Cas9 system) [119]. In general, by modulating various circRNAs identified to be involved in the differentiation or homeostasis of bone cells or in bone remodeling, it is possible to counteract bone pathologies. For instance, in bone marrow mesenchymal stem cells (BMSCs) obtained from patients with bone nonunion, the circRNA has_circ_0074834 was notably downregulated [120]. This circRNA was found to regulate *ZEB1* and *VEGF* via miR-942-5p. Its overexpression, obtained by using Lentivirus, was shown to enhance the osteogenic differentiation of BMSCs in vivo and promote bone regeneration in a mouse model of bone defects [120].

Additionally, advancements in nanoparticle-based drug delivery systems and tissue-specific targeting strategies hold promise for improving the efficacy and safety of circRNA-based therapies in different disorders.

Unfortunately, to date, these therapeutic approaches have solely been investigated in preclinical studies and there are no validated clinical studies where circRNA-based therapies have been applied to bone diseases. The primary limitations arise from off-target gene silencing, non-specific tissue or cell type targeting, and the immunogenicity of synthetic circRNAs. Thus, several challenges need to be addressed before the therapeutic potential of circRNA-based interventions can be fully realized.

## 5. Perspective

The exploration of circRNAs within the field of bone biology holds immense promise for future advancements. Firstly, gaining a comprehensive understanding of circRNA function and regulation in bone remodeling processes could unveil novel molecular pathways crucial for maintaining skeletal integrity and homeostasis. This knowledge could lead to the development of targeted therapeutic interventions for various bone disorders, ranging from osteoporosis to osteosarcoma. Secondly, the identification of circRNAs as potential biomarkers for bone-related diseases could revolutionize diagnostic approaches, enabling earlier detection and more accurate prognostic assessments. Furthermore, since osteogenesis and osteoclastogenesis are processes characterized by sequential phases during maturation, it is important to understand the modulation and role of circRNAs at each phase and how they impact specific molecular pathways throughout the differentiation process. Moreover, as sequencing technologies evolve and bioinformatics tools become more sophisticated, the discovery of new circRNAs and elucidation of their intricate interactions within bone cells may further enhance our understanding of skeletal biology, offering new avenues for tailored therapeutic strategies. In addition, analyzing the various circRNAs involved in bone pathologies, we did not find similarities among the different pathologies. However, given their ability to regulate gene expression at various levels and to act as sponges for various types of miRNAs, even if different, they might share common targets and therefore be associated with different pathologies. By exploring whether diverse circRNAs share common targets despite their differences, we may gain valuable insights into the underlying mechanisms of bone disorders and the role of circRNAs in their pathogenesis. Certainly, this field of research would represent a future research direction and a challenge. Ultimately, by delving deeper into the role of circRNAs in bone health and pathology, researchers can make significant contributions to the advancement of musculoskeletal medicine, thus improving patient outcomes and quality of life.

## 6. Conclusions

**Figure 1:**
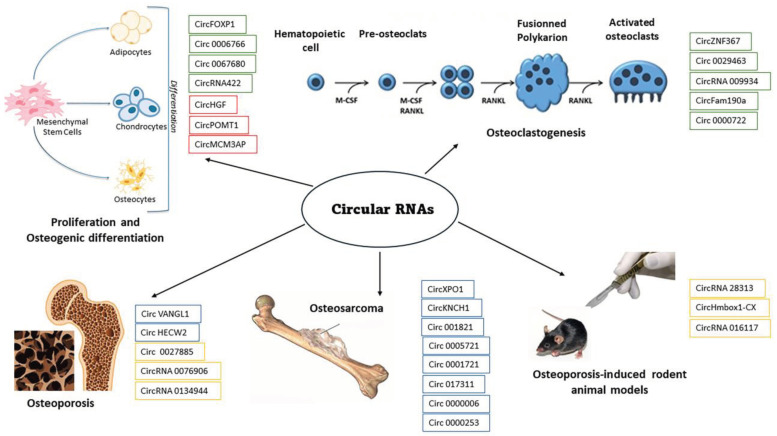
The scheme shows the effects of different circular RNAs that play a regulatory role in bone cell proliferation and differentiation in the upper panel. The boxes in green show the circular RNAs with a stimulatory effect and in red those with an inhibitory effect. The lower panel shows the effect on bone diseases (osteoporosis, osteosarcoma) and osteoporosis-induced animal models (OVX mice and GIOP rats). The blue boxes show the circular RNAs that induce increased disease and the orange boxes show those that control and reduce disease progression.

Circular RNAs exert a regulatory role on bone cell proliferation and differentiation and their alterations are involved in bone diseases (Figure 1). The dysregulation of circRNAs involved in osteogenesis or osteoclastogenesis is especially associated with impaired bone formation due to altered bone related cellular signaling activity. Moreover, circRNAs may serve as potential biomarkers for bone diseases, allowing for an early diagnosis or prognosis and risk stratification and the monitoring of disease progression. Additionally, circRNAs represent promising therapeutic targets for the management of bone disorders, offering novel insights into the molecular mechanisms underlying bone homeostasis and pathology. Harnessing the regulatory functions of circRNAs holds great potential for developing innovative therapies that can modulate bone metabolism, promote tissue regeneration, and improve clinical outcomes in patients with osteoporosis, bone fractures, and bone cancers. However, significant challenges must be addressed to unravel the complex roles of circRNAs in bone biology and to advance circRNA-based therapeutic strategies. Overcoming these challenges will pave the way for the development of personalized treatments and precision medicine approaches in bone medicine.

## Figures and Tables

**Table 1:** A partial list of circRNAs involved in osteogenesis.

circRNAs	Function	References
circ-DAB1	Promotes cell proliferation and osteogenic differentiation via the NOTCH/RBPJ axis	[52]
circ_0076906	Induces osteogenic differentiation via miR-1305/osteoglycin pathway	[53]
circ_0001795	Promotes the osteogenic differentiation via circ_0001795/miR-339-5p/YAP1 axis	[54]
circRNA_0024097	Promotes osteogenesis through miRNA-376b-3p/YAP1 axis	[55]
circ_0006859	Inhibits osteogenic differentiation by targeting miR-642b-5p/miR-483-3p	[56]
circRBM23	Regulates the switch between osteogenesis and adipogenesis by sponging miR-338-3p	[57]
circ-3626	Promotes osteogenesis by via miR-338-3p/Runx2 axis	[7]
Circ-FK501	Promotes osteogenic differentiation via microRNA-205-5p/RUNX2 axis	[58]
hsa-circ-0107593	Inhibits osteogenic differentiation via miR-20a-5p/SMAD6 signaling	[59]
Circ_0006873	Inhibits osteogenic differentiation via miR-20a-/SMURF2 signaling	[60]
Circ_0114581	Promotes osteogenic differentiation via the MiR-155-5p/HNRNPA3 axis	[61]
Circ_0001825	Promotes osteogenesis via miR-1270/SMAD5 axis	[62]
Circ_0036872	Promotes osteogenesis via miR-143-3p/IGF2 axis	[63]
CircZNF367	Inhibits osteogenic differentiation via reducing HuR-mediated mRNA stability of LRP5	[64]
Circ_C4orf36	Promotes osteogenesis by regulating VEGFA	[65]
Circ-Sirt1	Promotes osteogenesis by activating Sirt1 and Wnt/β-catenin pathway	[66]
